# HIV-Infected Individuals Who Delay, Decline, or Discontinue Antiretroviral Therapy: Comparing Clinic- and Peer-Recruited Cohorts

**DOI:** 10.3389/fpubh.2014.00081

**Published:** 2014-07-16

**Authors:** Marya Gwadz, Elizabeth Applegate, Charles Cleland, Noelle Regina Leonard, Hannah Wolfe, Nadim Salomon, Mindy Belkin, Marion Riedel, Angela Banfield, Lisa Sanfilippo, Andrea Wagner, Donna Mildvan, The Heart to Heart Collaborative Research Team

**Affiliations:** ^1^Center for Drug Use and HIV Research (CDUHR), New York University, College of Nursing, New York, NY, USA; ^2^Spencer Cox Center for Health, Mount Sinai St. Luke’s-Roosevelt Hospital Center, New York, NY, USA; ^3^Peter Kruger Clinic, Mount Sinai Beth Israel Medical Center, New York, NY, USA; ^4^School of Social Work, Columbia University, New York, NY, USA; ^5^Department of Infectious Diseases, Mount Sinai Beth Israel Medical Center, New York, NY, USA

**Keywords:** HIV, barriers, antiretroviral, African-American, Latino, discontinue, delay, decline

## Abstract

A substantial proportion of persons living with HIV/AIDS (PLHA) delay, decline, or discontinue antiretroviral therapy (ART) when it is medically indicated (40–45%), largely African-Americans and Latinos/Hispanics. This study explores the feasibility of locating PLHA, who are not on ART (PLHA-NOA) through clinics and peer-referral; compares the two cohorts on multi-level barriers to ART; and examines readiness to initiate/reinitiate ART, a predictor of treatment outcomes. We recruited adult HIV-infected African-American and Latino/Hispanic PLHA-NOA through HIV hospital clinics and peer-referral in 2012–2013. Participants were engaged in structured 1-h assessments with reliable/valid measures on barriers to ART. We found that recruitment through peers (63.2%, 60/95) was more feasible than in clinics (36.8%, 35/90). Participants were 48.0 years old and had lived with HIV for 14.7 years on average, and 56.8% had taken ART previously. Most (61.1%) were male and African-American (76.8%), and 23.2% were Latino/Hispanic. Peer-recruited participants were older, had lived with HIV longer, were less engaged in HIV care, and were more likely to have taken ART previously. The cohorts differed in reasons for discontinuing ART. Levels of ART knowledge were comparable between cohorts (68.5% correct), and there were no differences in attitudes toward ART (e.g., mistrust), which were in the neutral range. In bivariate linear regression, readiness for ART was negatively associated with physician mistrust (*B* = −10.4) and positively associated with self-efficacy (*B* = 5.5), positive outcome expectancies (*B* = 6.3), beliefs about personal necessity of ART (*B* = 17.5), and positive internal norms (*B* = 7.9). This study demonstrates the feasibility of engaging this vulnerable population through peer-referral. Peer-recruited PLHA evidence particularly high rates of risk factors compared to those in hospital clinics. Interventions to support ART initiation and continuation are sorely needed for both subgroups.

## Introduction

The proportion of persons living with HIV/AIDS (PLHA) who initiate antiretroviral therapy (ART) in a timely fashion has increased in recent decades (1, 2). Yet a substantial proportion of PLHA is not taking ART when it is medically indicated, because they decline or delay ART when it is offered, discontinue ART, or do not otherwise have good access to ART regimens (3, 4). We refer to this population of PLHA not on ART when it is medically indicated as “PLHA-NOA.” The individual, community, and societal-level consequences of PLHA not gaining access to ART are grave, and include high rates of morbidity, reduced quality of life, earlier mortality, viral resistance (when ART is discontinued), increased risk of transmission of HIV to others, and high health care costs (5–9).

Studies characterizing rates of diagnosis with HIV, linkage to and retention in care, ART uptake, and viral suppression, called the “HIV continuum of care” or “treatment cascade,” highlight the very serious problem of PLHA-NOA. A 2012 Centers for Disease Control and Prevention (CDC) report described a number of gaps: of the 1.1 million Americans living with HIV, 63% are not retained in care; 67% have not been prescribed ART, and only one-quarter have suppressed viral load (3). Based on these CDC data, we conservatively estimate that 40–50% of PLHA in the US have unmet need for ART, mainly those who are also not well engaged in care. These high estimates of PLHA-NOA are supported by data on ART discontinuation, where up to 40–50% of those who initiate ART discontinue their regimens at least once within a year (10–12).

The majority of PLHA in the US are from African-American and Latino/Hispanic backgrounds (approximately 60%), and likewise, the majority of PLHA-NOA is from racial/ethnic minority backgrounds (3, 13). Indeed, the racial and ethnic background of PLHA-NOA is relevant because barriers to ART tend to vary across racial/ethnic groups, and interventions to reduce barriers to health can be culturally targeted to the factors with the greatest relevance to various subgroups of PLHA (14, 15).

In contrast to the large literature on ART adherence, little is known about the factors that drive the problem of PLHA-NOA. In the next section, we consider factors believed to contribute to discontinuation, delay, and decline of ART, and poor access to ART. In light of the complexity of the problem of PLHA-NOA, the present study was guided by the theory of triadic influence, a multi-level social-cognitive theory that examines three “streams of influence” on health behavior: individual/attitudinal, social, and structural (16).

At the individual/attitudinal level of influence, poor understanding of HIV treatment as well as misconceptions about ART appears to contribute to PLHA-NOA(17). PLHA may not perceive the need for ART, particularly when they feel healthy (18). African-American and Latino/Hispanic PLHA evidence a number of HIV- and health care-related attitudes and beliefs rooted in culture and U.S. history. Specifically, these beliefs are partly grounded in the history of past abuses of people from racial/ethnic minority backgrounds by medical research, the medical establishment, and larger society, which have been described as resonating with present-day exclusion, discrimination, and structural racism (13, 19–21). The Tuskegee Syphilis study is perhaps the most emblematic example of past abuses, and this and other examples of maltreatment contribute to fear and distrust, not only of ART, but also the HIV care system, health care providers, and other larger societal structures (22, 23). Further, negative perceptions of ART, particularly fear of side effects, are common and can impede acceptance of ART (24–26), as do negative outcome expectancies, that is, the belief ART is ineffective and/or toxic (26). Moreover, low self-efficacy for managing and adhering to ART may reduce ART initiation (24, 25, 27). These attitudes and intentions appear to interact to contribute to a belief that ART is unnecessary or even harmful that it cannot be tolerated or successfully managed, and/or create fears that one is not “ready” to manage an ART regimen (24, 28). Indeed, a number of studies have argued that readiness for ART is an important latent construct that influences both adherence and treatment outcomes, which can therefore be targeted in behavioral interventions to improve ART outcomes (29, 30). Yet fears and negative health beliefs do not preclude interest in ART or even ART initiation. PLHA, including those from racial/ethnic minority backgrounds, can simultaneously evidence curiosity about and willingness to initiate treatment options, along with fear and negative health beliefs (31, 32).

Persons living with HIV/AIDS’ health status can impede uptake of ART. PLHA face challenges accessing HIV health care (3, 33) and sub-optimal engagement in care is common (3, 5). Those attending fewer health care visits are less likely to initiate ART (34), suggesting that those with poor access to care are less likely to use ART, those who do not wish to initiate ART elect to attend fewer health care appointments in order to avoid such discussions with providers, and that barriers to ART also impede access to care (35). Moreover “competing priorities” interfere with initiation of ART, primary among them are alcohol and non-injection drug use problems (36, 37), injection drug use in some studies, mental health symptoms, and unstable housing (17, 27, 38–40). Yet competing priorities, while serious, do not necessarily preclude individuals from initiating and sustaining ART with high adherence. Past studies have shown these types of barriers can be ameliorated through interventions, and that among populations with competing priorities, rates of ART initiation with high adherence improve with clinical care, behavioral interventions, and support (36).

Social-level barriers include fear of HIV-related stigma that might arise if others were aware of their HIV diagnosis. Moreover, PLHA who perceive negative peer norms regarding ART, that is, that their peers tend to avoid ART or view ART negatively, or would view the PLHA negatively if he/she took ART, may be less likely to initiate and sustain these regimens (41, 42). PLHA’s relationships with health care providers play a critical role in ART initiation. National HIV treatment guidelines recommend that providers evaluate and manage factors that might impede adherence to ART (43). Yet some studies suggest that providers may delay recommending ART to patients because of concerns that patients will be unable to benefit from or adhere to ART (4, 25, 26, 44). Wong and colleagues (4) found that African-Americans initiate ART later than Whites regardless of providers’ attitude toward prescribing ART, but that provider beliefs led to delayed prescribing of ART to Latinos/Hispanics, women, and those with low socioeconomic status (4). The problem of PLHA-NOA is complicated by the fact that some patients are hesitant to tell their providers that they are not taking ART, for example in cases when providers have prescribed ART and assume patients have initiated it (45).

Structural barriers are features of the external environment (economic, geographic, policy, organizational or other) that limit individuals’ options (46). Aspects of the HIV care setting including its location, structure, policies, and quality of care can promote or impede access to ART (47, 48). While few studies have compared the initiation of ART, adherence, or retention in care between patients in comprehensive hospital HIV clinics vs. those in community care settings, recent work suggests that rates of initiation of ART are similar between the two settings, although community-based clinics may see patients earlier in their HIV disease as a result of outreach efforts (49, 50). Yet another recent study suggests that patients in community-based clinics have better health care retention but those in hospital-based clinics are more likely to be virally suppressed (51). Thus the setting where PLHA receive care may influence patterns of ART initiation. A second structural barrier to ART may be the short health care encounter, which is not well designed to overcome multiple, intersecting barriers to ART (40). Last, in some locations, vulnerable PLHA are offered the chance to sell medications back to a pharmacy, an illegal activity that thereby decreases uptake of ART (51).

The HIV continuum of care models indicate that PLHA-NOA can be found in clinic settings (3). Yet little is known about strategies to seek out PLHA-NOA who do not regularly attend clinic visits. Because PLHA tend to be socially linked (52), peer-recruitment methods may hold promise for seeking out and engaging PLHA-NOA, including those with less frequent involvement in care (52, 53). Thus one aim of the present study was to explore the feasibility of both clinic- and peer-based strategies for locating and engaging PLHA-NOA into a research study on barriers to ART initiation and continuation.

This is an exploratory study of an under-studied population, PLHA-NOA, focused on the population of African-American and Latino/Hispanic individuals PLHA who comprise the majority of this vulnerable group. The aims of the study were to extend past research on PLHA-NOA that has focused mainly on clinic settings and (1) explore the feasibility of recruiting PLHA-NOA from HIV hospital clinics compared to peer-referral, and gain insights to refine recruitment procedures for future research; (2) describe the clinic and peer-recruited cohorts and compare them with respect to socio-demographic, background, and health characteristics, including reasons for delaying/declining or discontinuing ART, as appropriate; (3) compare individual/attitudinal-, social-, structural-level factors hypothesized to impede ART initiation between the two groups; (4) identify factors that predict “readiness” to initiate ART, including whether cohort (clinic vs. peer-recruited) and past use of ART are associated with readiness to initiate ART; and (5) explore whether those who have taken ART in the past differ from those who have never taken ART on background, health, and other factors. We hypothesized that it would be more feasible to locate and engage PLHA-NOA in clinic settings than through peers, because they have a prior connection to the setting, which could facilitate linkage to the study. Because little is known about the barriers to ART for PLHA-NOA, we do not present hypotheses regarding how clinic and peer-recruited cohorts may differ with respect to these factors.

## Materials and Methods

Participants were 95 individuals recruited in HIV hospital clinics (*N* = 35) and through peer-referral (*N* = 60), who were medically eligible for ART but not taking ART, as part of an intervention development study on increasing ART initiation in this group. The clinic-recruitment study component was located in two large hospital-based HIV clinics in New York City, each serving between 1000 and –4000 PLHA, primarily those from African-American and Latino/Hispanic backgrounds, where prior to the study an estimated 10% of the patient population had delayed, declined, or stopped ART when it was medically indicated. The peer-referral component, described below, was located at a project field site. Recruitment procedures for the two cohorts differed in a modest number of respects, as we describe below, although eligibility criteria were the same. Procedures were approved by the Institutional Review Boards at the two collaborating hospitals and New York University.

### Rationale for structure of procedures

**Figure 1:**
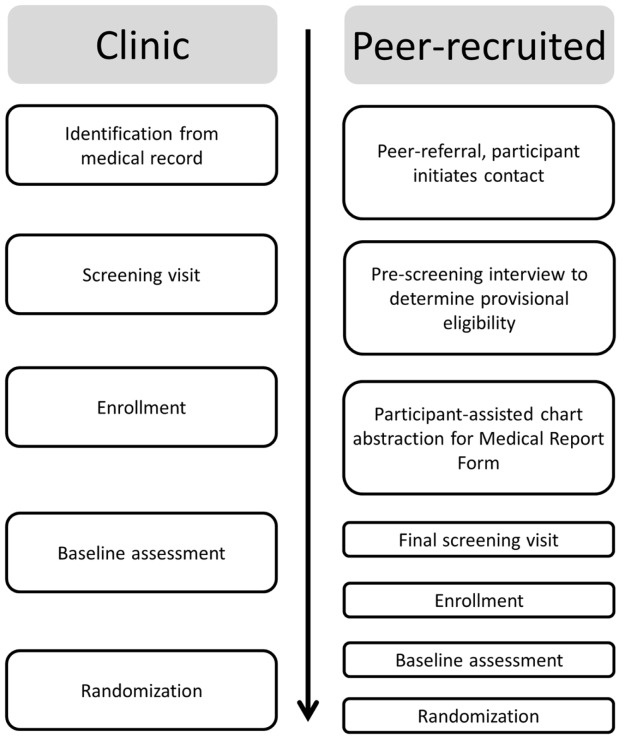
**Enrollment procedures**.

As described above (see Introduction), PLHA-NOA experience numerous barriers to ART. As a result, PLHA may not fill ART prescriptions written for them, may fill them but not take ART, may sell ART to a pharmacy, or may stop taking ART, but may not feel comfortable disclosing their ART decisions to providers (45). Thus, although providers generally discuss ART with patients and monitor clinical indicators of treatment effects at regular intervals – assuming patients present for care – providers may not know for certain if an individual is taking ART. This complicates efforts to determine whether an individual is taking ART for the purposes of research: participants may be motivated to obscure the fact that they are actually taking ART to enroll in the study, and on the other hand, participants who are not taking ART may be hesitant to disclose this fact, particularly in cases where the provider believes they are taking ART. Thus, in light of potentially conflicting information from providers, the medical record, and patients, in at least a minority of cases, procedures were developed to attempt to tease out which patients were not on ART and who therefore met this study inclusion criterion, as we describe below (see Figure 1).

### Recruitment and enrollment

#### Clinic sample

##### Extraction of health information from the medical record

From the medical record, the HIV clinics identified participants (1) aged 18 years or older, (2) African-American or Latino/Hispanic, (3) HIV infected for ≥6 months, (4) medically eligible for ART for ≥3 months, (5) had seen a provider at the clinic at least once in the past year, (6) last CD4 count <500 cells/mL, (7) able to conduct research activities in English, and (8) with indications that the patient may not be taking ART including (a) medical record indicates patient is not on ART (e.g., ART refused, ART not prescribed) or (b) patient has been prescribed ART for at least 3 months but has detectable viral load (>1000 copies/ppmL), and (9) patient does not have any condition that in the opinion of the primary care provider would interfere with provision of informed consent or make it unsafe for the patient to participate in this study. Patients meeting these criteria were included on a “recruitment roster.” Determination of ART status (i.e., never took ART or took ART in the past but on fewer than 60 days in the past 6 months and not at all in the past 30 days) tool place in a subsequent step, as described in the next section.

##### Screening visit

Patients on the recruitment roster were either met in person at the time of their next clinic appointment or contacted by phone by a clinic staff member (depending on the policies of the clinic), and invited to a screening visit, called a “health check up” (HCU). Participants gave signed informed consent before participating in the screening visit. The screening visit had two main goals: (1) engagement and relationship building and (2) evaluation of ART status to determine whether the patient was eligible for the study. The screening interview alternated between structured assessment instrument segments using the computer-assisted personal interview format (CAPI), and open discussion of pre-specified questions health and ART issues. Thus we also collected other demographic and health information, which were included in the research record if the patient enrolled in the study. The open-ended discussion component was guided by the motivational interviewing approach (54) to encourage frank discussion of sensitive subjects (e.g., medication selling, having stopped ART but not yet having told the provider), to convey respect for autonomy, and reduce fear and social-desirability bias (54). With respect to the ART inclusion criterion, participants were coded as (10a) never took ART; (10b) took ART in the past but not in the past 6 months; (10c) took ART in the past but on fewer than 60 days in the past 6 months and not at all in the past 30 days (because PLHA may initiate ART but stop after a brief period); and (10d) took ART on more than 60 days in the past 6 months or took ART in the past month. Those in categories 10a–10c were eligible for the study and those coded as category 10d were ineligible and were referred to adherence counseling or their provider, as appropriate. Those found eligible based on all inclusion criteria assessed from the medical record, provider, and this screening interview were then invited to participate in the larger research study. The screening visit took place at the HIV clinic sites. Participants received compensation of $30 plus funds for local transportation for this visit. In keeping with the exploratory nature of the study, when the medical record and participants’ reports were discrepant with respect to ART initiation (namely, the participant stated that he/she was not taking ART but the provider or medical record indicated he/she most likely had initiated it), participants were enrolled into the study. Participants gave consent for data from the medical record data, which were recorded on a Medical Report Form, to be included in the research record. Otherwise, they were destroyed.

##### Study enrollment and baseline assessment

After providing signed informed consent for remaining study activities, participants found eligible for the study engaged in a structured baseline assessment on HIV knowledge, a range of attitudes toward and beliefs about ART, and health care use patterns on a computer, lasting approximately 1 h. The assessment was conducted in both computer-assisted audio interviewing (CAPI) and audio, computer-assisted self-interviewing (ACASI) formats. Assessments took place at the HIV clinics. Participants received $20 and fare for local public transportation for their time. Clinic- and peer-recruited participants completed the same set of measures, which focused on the lifetime and past 6 months. The amount of time to enroll participants into the clinic-recruited cohort was 3–6 weeks, with participants typically being enrolled within 4 weeks.

#### Peer-recruited cohort

##### Peer sampling method

Participants were recruited using a chain-referral peer sampling method (53). Initial recruiters (*N* = 88) were drawn from a pre-existing recruitment registry of HIV-infected individuals maintained by the research study team. Individuals in the recruitment registry who met inclusion criteria were also eligible for the study (*N* = 9 enrolled). We also provided other peer-recruited study participants with the opportunity to recruit their own peers. Recruiters were given up to 10 coded recruitment coupons and instructed to provide these coupons to HIV-infected individuals who they knew by name or face, who may not be on ART or engaged in health care (a proxy for delaying, declining, or discontinuing ART). To protect confidentiality, peers contacted the study directly to be screened for eligibility. Recruiters received compensation of $10 for each peer who contacted the study and was screened. We also accepted direct referrals from participants who had heard about the study in the community, and recruited directly at number of community-based organizations. The order of study activities for the peer-recruitment cohort differed in some respects from those described above, because we did not have a pre-established relationship with the health care providers of these participants, and did not have access to their medical records at the time the participant presented to the study, as we describe below.

##### Brief pre-screening health interview to determine provisional eligibility

Peer-referred individuals contacted the study, and were scheduled for a brief pre-screening health interview conducted in person at a project field site. Participants gave verbal informed consent for this activity. Inclusion criteria for this first stage of eligibility included (1) aged 18 years or older, (2) African-American or Latino/Hispanic, (3) HIV infected for ≥6 months, (4) able to conduct research activities in English, (5a) never took ART or (5b) took ART in the past but on fewer than 60 days in the past 6 months and not at all in the past 30 days. To meet the CD4 criterion we assessed whether (6a) last CD4 ≤ 500 cells/mL or (6b) the participant had not had CD4 tested in past year or (6c) he/she did not know his/her CD4 count. These criteria were assessed by self-report and were later confirmed by the medical record where possible. HIV status was confirmed with medical documentation prior to enrollment (examples include test results from a lab or testing site, local HIV benefits eligibility card). Participants who meet these criteria were provisionally eligible to enroll in the study. They received compensation of $15 and fare for local transportation for this brief interview.

##### Participant-assisted chart abstraction for medical report form

As noted above (see Extraction of health information from the medical record), two inclusion criteria are reported by the participant’s health care provider (#4 – medically eligible for ART for ≥3 months; #9 – whether the patient had any condition that would interfere with provision of informed consent or make it unsafe). Peer-recruited participants provisionally eligible at screening provided signed informed consent for the project to have the health care provider complete a Medical Report Form with these and other inclusion criteria drawn from the medical record. Alternately, participants could ask the provider themselves to complete the form. Participants received compensation of $15 for having the Medical Report Form completed. In keeping with the exploratory nature of the present study, participants not linked to health care (defined as not engaging in any HIV-related health care visits in past year) could remain in the study, and were referred to HIV care during the study.

##### Screening visit to establish eligibility

As described above (see Screening visit), participants engaged in a screening visit with structured and open-ended components to determine eligibility with respect to ART status. Participants received compensation of $30 for this session, plus fare for local transportation.

##### Enrollment and assessment

Participants who met the inclusion criteria provided signed informed consent to be enrolled in the study, and participated in the structured baseline interview as described above (see Study enrollment and baseline assessment). The amount of time to enroll participants into the peer-recruited cohort was 1–8 weeks, with participants typically being enrolled within 6 weeks.

### Measures

#### Socio-demographic, background, and health characteristics

We assessed socio-demographic characteristics such as age, sex, race/ethnicity, sexual minority status, and employment status with a structured measure (55). We assessed HIV history including year of first HIV diagnosis, time between diagnosis and engagement in health care, current health status (at a level of good or better), nadir and most recent CD4 levels, most recent viral load levels, and ART history with a measure from the HIV cost and services utilization study (HCSUS) (56). HIV-related symptoms were assessed with the symptoms distress module (57). The module uses a five-point scale, which is summed to characterize the extent of symptom distress experienced (Cronbach’s α = 0.87). HIV care utilization in the past 6 months was assessed with items from the service utilization battery (58). We assessed 11 reasons for discontinuing ART using a scale developed by Johnson and colleagues (yes/no) (18).

#### Individual/attitudinal influences

##### HIV treatment knowledge

Treatment knowledge was assessed on a reliable 14-item three-point scale (true, false, and not sure, Cronbach’s α = 0.76). Treatment knowledge was scored as the number of items answered correctly (range 0–14) (59).

##### Mistrust

General medical mistrust, that is, mistrust of the *healthcare system in general*, was assessed with a seven-item assessment using a five-point Likert-type scale (strongly disagree to strongly agree) (Cronbach’s α = 0.72). After reverse coding of one item, the total score was the average across the items, which ranged from 0 to 4, with a higher number indicating greater mistrust (60). HIV and ART mistrust was assessed with a reliable (Cronbach’s α = 0.84) 10-item measure that assesses attitudes toward healthcare organizations that provide HIV care and HIV medication on a five-point Likert-type scale (strongly disagree to strongly agree) (61). After reverse coding of two items, the total score was the average across items, which ranged from 0 to 4, with a higher number indicating greater mistrust. Mistrust in one’s health care provider was assessed by the trust in physician scale (62), an 11-item measure assessing measuring dependability, confidence, and confidentiality of information on a five-point Likert-type scale (strongly disagree to strongly agree) (Cronbach’s α = 0.88). All items were coded such that a higher number indicated greater mistrust. The total score was the average across items, which ranged from 0 to 4.

##### ART health beliefs

Antiretroviral therapy taking self-efficacy was assessed with a 17-item highly reliable measure that uses a 10-point Likert scale, with higher scores indicating greater HIV medication-taking self-efficacy (Cronbach’s α = 0.95) (63). ART outcome expectancies, in this case, are the participant’s confidence that ART will have beneficial health and quality of life outcomes if taken, were assessed on a nine-item measure that uses a 10-point Likert scale with higher scores indicating more positive ART outcome expectancies (Cronbach’s α = 0.95) (63). Further, the beliefs about medication questionnaire (BMQ) (64) is a 13 item scale comprising two subscales measuring beliefs about medications, revised to assess beliefs about ART: personal necessity for ART (e.g., my health in the future will depend on these medications, Cronbach’s α = 0.88) and concern/fear about effects of ART (e.g., the idea of taking HIV medications worries me, Cronbach’s α = 0.68). Participants indicate that the degree of agreement with individual statements about ART on a five-point Likert scale ranges from 1 = strongly disagree to 5 = strongly agree. Scores obtained for the individual items within each scale are summed to give a scale score.

##### “Readiness” to take ART

“Readiness” to take ART at the present time was assessed using two items, modeled on the approach developed by Rollnick (65): the perceived importance of taking ART and confidence that he/she could take HIV medication every day, as prescribed, if he/she so desired (65). Both were assessed on a 0–10 scale and these two ratings were multiplied to create the “readiness” variable, leading to a range from 0 to 100, where higher scores indicate greater readiness for ART.

#### Social factors

##### ART-related stigma

Concern about stigma associated with ART was assessed with three items drawn from the Patient Medication Adherence Questionnaire (66) (e.g., I don’t want people to see me take my HIV medicines), assessed on a three-point scale (disagree, not sure, and agree). Each item was scored from 1 to 3, and the total score was the average across items, with a higher score indicating greater stigma (Cronbach’s α = 0.73).

##### Social norms supportive of ART

Egocentric social norms supportive of ART were assessed using a single item tapping into the extent to which social network members living with HIV are perceived as favorable toward or currently taking ART using a seven-point Likert-type scale ranging from none to almost all social network members (how many of the people you know who are living with HIV or AIDS would consider taking HIV medication?) (67). External injunctive social norms, the extent to which participants perceive close friends and family as encouraging or supporting the participant to take ART, were assessed with a single item using a seven-point Likert-type scale ranging from none to almost all social network members (how many of your close friends and family would support you if you started HIV medication?) (67). Internal norms supportive of ART (how you feel about things others may do?) were assessed with a single item using a four-point Likert-type scale (strongly disagree to strongly agree) that was reverse coded (I think people who take HIV medications are making a mistake.) (67).

#### Structural factors

##### Received care in a hospital clinic

Using a single item we assessed whether participants received care in a hospital HIV clinic, which tend to be larger and provide a comprehensive set of high-quality health, mental health, and social services, vs. other settings (private practice, community setting, and emergency room).

##### Satisfaction with health care

Satisfaction with health care was assessed with the Client Satisfaction Survey (68), a four-item measure that uses a four-point Likert scale. Individual items were scored from 0 to 3, with higher numbers indicating greater satisfaction. The total score was the average across items (Cronbach’s α = 0.85). Satisfaction with care could be considered an individual/attitudinal-level variable, but was used as a proxy for setting quality in this study.

##### Selling ART

Participants were asked, “People have told us that in tough times, they have sold their HIV medication to others in order to pay for necessities. In your whole life, have you ever had to sell your HIV medications?” Participants were also asked if they had sold ART over the past 6 months. Both items were scored as yes/no.

### Missing data

Missing data were handled by the multiple imputation method, which is more powerful and less biased than *ad hoc* methods of handling missingness (e.g., listwise deletion). This approach assumes that data are missing at random (MAR), that is, MAR conditional on values observed. Missing data were imputed 100 times using a chained equation approach (69), as implemented by the R Core Team (70) MICE package (71). Estimates and standard errors from complete-data analyses of these 100 imputed datasets were combined into a single inference (point estimate, standard error, and confidence interval) using the approach described by Rubin (72). Details on the extent of missing data are provided in the tables. The variables with the largest percentage of missing data included medical records-based viral load (52%) and CD4 (36%), which we were unable to obtain from health care providers or medical institutions for some in the peer-recruited cohort. Across all other study variables, less than half of all variables had any missing data, and data were missing for at most 8% of participants (the “egocentric norms supportive of ART” measure).

### Data analysis

Bivariate linear regression was used to estimate associations between potential predictors and ART readiness. Then, multiple regression analysis was used to examine, among the variables with a significant bivariate association with ART readiness, which variables also had a significant unique association with ART readiness. The *R* statistical computing environment was used for all analyses (70).

## Results

### Feasibility of enrolling in clinics and through peer-referral

#### Clinic cohort

The total number of patients at the two HIV clinics was 4758, of which 206 (4.3% of the clinic population) were not on ART and 148 met preliminary eligibility criteria. These 148 individuals (3.1% of the clinic population) were targeted for screening and, if eligible, enrolled. Of these, we were unable to contact 99 (66.9%), often because participants did not present for appointments at the scheduled times and we were therefore unable to recruit them. Further, eight individuals declined to participate (5.4%), providers did not approve participation in six cases (4.1%); and eight were found to be ineligible in the screening meeting (5.4%). A total of 35 (23.7% of the 148 found preliminarily eligible) were found eligible and were enrolled.

#### Peer-recruited cohort

A total of 135 were pre-screened through peer-referral (*N* = 108), the recruitment registry (*N* = 15), and directly recruitment from community-based organizations (*N* = 12). Most (*N* = 119; 88.2%) were found preliminarily eligible based on self-report data. Next, of these 119 potential participants, we obtained medical report forms to confirm inclusion criteria from 55 participants (46.2%). We found that 26 were ineligible (*N* = 22, CD4 > 500; *N* = 3 provider reported patients was taking ART and patient confirmed in the screening meeting; *N* = 1 provider did not consent to patient participating). The remaining 29 were found eligible (*N* = 11 provider reported the patient was on ART but the patient reported not taking it; *N* = 8 provider was unsure if the patient was taking ART; *N* = 10 provider reported patient was not on ART). For 34, the medical report form was waived because the participant reported not having seen a health care provider in the past year. A total of 60 peer-recruited participants were enrolled, 50.4% of the 119 found preliminarily eligible.

### Socio-demographic and health characteristics

Socio-demographic and other characteristics of the sample are described in Table 1. Approximately 60% of the sample was male and the mean age was 48.2 years (SD = 8.88 years). More than two-thirds (76.8%) of participants were African-American, and the remainder was Latino/Hispanic (23.2%). More than a third (39.0%) was a “sexual minority” (lesbian, gay, or bisexual). Few were currently employed (16.8%), but most had a high school diploma or equivalent (66.3%). Those in the peer-recruited cohort were significantly older and less likely to be employed, but did not differ from their clinic-recruited peers on other socio-demographic characteristics. Participants had been diagnosed with HIV for 14.7 years on average (SD = 8.73 years). At first diagnosis, 40% had delayed entering care for more than 6 months. The most recent CD4 count from the medical report forms was 302.8 cells/mL on average (SD = 138.9 cells/mL) and log_10_ viral load was 3.49 (SD = 1.36). Self-reported CD4 were comparable to the medical record data: 343.06 cells/mL (SD = 128.57 cells/mL), as was viral load. About half (56.8%) had taken ART in the past. Almost all (99.0%) reported that their provider had recommended that they take ART over their lifetimes. About three-quarters (71.6%) had attended one or more HIV care appointments in the past 6 months. Rates of HIV-related symptoms were modest on average (18.03 on average on a 0–72 scale; SD = 14.46). Compared to their clinic-recruited peers, the peer-recruited cohort exhibited worse health outcomes on almost every index, as shown in Table 1.

**Table 1:** **Socio-demographic, background, and health characteristics**.

	Clinic (*N* = 35) Mean (SD)/%	Peer-referred (*N* = 60) Mean (SD)/%	Total (*N* = 95) Mean (SD)/%	*p*
Age in years	44.8 (10.8)	49.9 (7.0)	48.0 (8.9)	*
Male sex	65.7	58.3	61.1	
African-American, not Latino/Hispanic	71.4	80.0	76.8	
Latino/Hispanic	28.6	20.0	23.2	
Sexual minority status (gay, lesbian, and bisexual)	48.6	33.3	39.0	
Currently employed	28.6	10.0	16.8	*
**HEALTH**				
Years since HIV diagnosis (self-report)	12.1 (9.1)	16.13 (8.2)	14.65 (8.7)	*
>6 months between HIV diagnosis and care appointment	25.7	48.3	40.0	*
Health self-rating “good” or better	77.1	61.7	67.4	
CD4 nadir/lowest CD4 cell count ever	246.0 (142.4)	136.6 (116.6)	173.6 (135.3)	**
Most recent CD4 (medical report form)	344.8 (128.6)	249.9 (135.4)	302.8 (138.9)	**
Most recent CD4 (self-report)	343.1 (127.1)	254.3 (143.4)	291.7 (143.0)	**
Most recent log_10_ viral load (medical report form)	3.9 (0.9)	2.9 (1.7)	3.5 (1.4)	*
Most recent log_10_ viral load (self-report)	3.9 (1.1)	3.2 (1.5)	3.4 (1.4)	*
Health care provider recommended ART (lifetime)	100.0	98.3	99.0	
Medical provider knows not taking HIV medications at this time	100.0	88.3	92.6	*
>1 HIV health care appointments in the past 6 months	88.6	61.7	71.6	**
Symptoms distress module (0–72)	16.9 (15.8)	18.7 (13.7)	18.0 (14.5)	
Ever taken ART	42.9	65.0	56.8	^§^

Categorical variables compared via Fisher’s exact test and other variables via independent samples *t*-test, without assuming equal variances. Data were missing for the following variables: CD4 nadir (24); recent CD4 report form (34); recent CD4 self-report (12); viral load report form (49); and viral load self-report (40). **p ≥ 0.01 level, *p ≥ 0.05 level, ^§^*p* ≥ 0.10 level.

### Reasons for ART discontinuation

As presented in Table 2, among those who had taken ART previously, the main reasons for participants’ discontinuing ART in the past were side effects (68.6%), “changed my mind” (62.0%), “life circumstances changed” (54.9%), difficulties with adherence (47.1%), substance use got in the way (45.1%), and “was not ready” (39.2%). About half (52.9%) reported other reasons for discontinuing ART in an open item question, which included the following: ART was taken only during pregnancy and the participant did not wish to continue, difficulty swallowing or dislike of pills, fear of long-term toxicity, stigma, felt healthy, did not wish to accept one had HIV, and medication selling. Peer-recruited participants differed from their peers in a number of respects: they were significantly more likely to report the following reasons for discontinuation than the clinic-recruited cohort: losing housing, life circumstances changed, substance use, changed their minds, and not being ready.

**Table 2:** **Reasons for ART discontinuation**.

	Clinic (*N* = 14) (%)	Peer-referred (*N* = 37) (%)	Total (*N* = 51) (%)	*P*
Difficulties with adherence	28.6	54.1	47.1	
Side effects	50.0	75.7	68.6	
Lost housing	0.0	24.3	17.7	*
Provider told me to stop	21.4	13.5	15.7	
Friends or family told me to stop	7.1	5.4	5.9	
Problems getting prescription filled	0.0	10.8	7.8	
Life circumstances changed	7.1	73.0	54.9	**
Substance use got in the way	14.3	56.8	45.1	*
Changed my mind	28.6	75.0	62.0	**
Was not ready	14.3	48.7	39.2	*
Other reason	78.6	43.2	53.0	*

Comparisons made by Fisher’s Exact test. Data were missing for the following variables: items 1–8, 10, and 11 (3); item 9 (4). **p ≥ 0.01 level, *p ≥ 0.05 level.

### Multi-level barriers to ART

#### Individual/attitudinal-level factors

Participants answered an average of 68.5% of items correctly, with no differences between cohorts (9.55 items correct on the knowledge scale, SD = 3.02 items) as shown in Table 3. Levels of general, HIV, and health care provider mistrust were moderate with no difference between cohorts. Mean scores were 2.08 (SD = 0.66), 2.36 (0.65), and 2.48 (0.7), respectively, corresponding to “neutral” on the five-point scale. Self-efficacy scores indicated that participants’ views on their abilities to manage ART were neutral (mean 6.52 on 1–10 scale, SD = 2.13), with no cohort differences. Similarly, outcome expectancies, that is, whether ART is beneficial, were in the positive range for both cohorts (mean = 7.12 on a 1–10 scale, SD = 2.53). Personal necessity for ART was moderate (mean = 2.08 on a 0–4 scale which corresponds to an answer of “neutral,” SD = 0.88). Concern about/fear of ART was also moderate (mean = 2.36 on a 0–4 scale which corresponds to an answer of “neutral,” SD = 0.63). “Readiness” to take ART was modest for both cohorts, with a mean score of 36.23 (SD = 34.38). The two cohorts were similar to each other on these individual/attitudinal factors as shown in Table 3 with two exceptions: the peer-referred cohort reported significantly higher personal necessity for ART, but lower concern about/fear of ART.

**Table 3:** **Knowledge of, attitudes toward, and access to ART**.

	Clinic (*N* = 35) Mean (SD)/%	Peer-referred (*N* = 60) Mean (SD)/%	Total (*N* = 95) Mean (SD)/%	*p*
**INDIVIDUAL-ATTITUDINAL FACTORS**				
HIV treatment knowledge (0–14)	9.3 (2.9)	9.7 (3.1)	9.6 (3.0)	
HIV medication distrust (0–4)	2.1 (0.7)	2.1 (0.6)	2.1 (0.7)	
General medical distrust (0–4)	2.3 (0.7)	2.4 (0.6)	2.4 (0.7)	
Physician distrust (0–4)	1.4 (0.7)	1.6 (0.7)	1.5 (0.7)	
ART self-efficacy beliefs (0–10)	6.8 (1.8)	6.3 (2.3)	6.5 (2.1)	
ART outcome expectancies (0–10)	6.9 (2.7)	7.3 (2.5)	7.1 (2.5)	
Personal necessity for ART (0–4)	1.8 (0.9)	2.2 (0.8)	2.1 (0.9)	*
Concern about/fear of ART (0–4)	2.5 (0.7)	2.3 (0.6)	2.4 (0.6)	*
“Readiness” for ART (0–100)	41.8 (36.5)	33.0 (33.0)	36.2 (34.4)	
**SOCIAL FACTORS**				
Stigma (1–3)	1.9 (0.9)	2.0 (0.6)	2.0 (0.7)	
Egocentric norms supportive of ART (0–6)	4.8 (1.5)	3.5 (1.8)	3.9 (1.8)	**
External injunctive norms supportive of ART (0–6)	4.6 (2.2)	4.7 (1.9)	4.7 (2.0)	
Internal norms supportive of ART (0–6)	4.1 (1.5)	4.3 (1.1)	4.2 (1.2)	
**STRUCTURAL FACTORS**				
Receive HIV care in a hospital clinic	100.0	31.7	56.8	**
Satisfaction with health care (0–3)	2.2 (0.6)	1.8 (0.8)	1.9 (0.7)	**
Ever sold ART	14.3	26.7	22.1	
Sold ART in the past 6 months	5.7	1.7	3.2	

Categorical variables compared via Fisher’s exact test and other variables via independent samples *t*-test, without assuming equal variances. Data were missing for the following variables: ART readiness (3); egocentric norms (8); external norms (4); internal norms (6); and client satisfaction (7). **p ≥ 0.01 level, *p ≥ 0.05 level.

#### Social-level influences

Both cohorts reported similar concerns about stigma as a barrier to ART (*X* = 1.99 on a 1–3 scale; SD = 0.72). Perceived social norms (egocentric, external, and internal) were also in the neutral range (3.9–4.7 on 0–6 scale). Peer-recruited participants had significantly more negative egocentric norms (suggesting that they view fewer of their peers as favorable toward ART), but did not differ on external or internal norms compared to the clinic-recruited cohort.

#### Structural-level influences

About half received HIV care in a hospital-based clinic: all of those in the clinic-recruited cohort (as expected) and 31.7% of the peer-recruited cohort. Satisfaction with care tended to be satisfactory (*X* = 1.94 on a 0–3 scale, SD = 0.7) but the peer-recruited cohort reported lower satisfaction compared to the clinic cohort. A total of 22.1% had ever sold medication in the past across the two cohorts.

### Differences between those who have discontinued ART and those who have never taken ART (ART naïve)

Compared to those who have never taken ART (that is, ART naïve), PLHA who had discontinued ART tended to be significantly older [mean = 49.9 (SD = 8.0) years vs. 45.5 (SD = 9.4) years, *p* ≤ 0.05]; had lived with HIV longer [mean = 16.9 (SD = 7.6) years vs. 11.6 (SD = 9.3) years, *p* ≤ 0.01]; had higher levels of HIV knowledge [mean = 10.4 (SD = 2.5) vs. 8.4 (SD = 3.3)]; had lower levels of concern about ART [mean = 2.1 (SD = 0.6) vs. 2.7 (SD = 0.5), *p* < 0.001]; and lower levels of perceived stigma [mean = 1.9 (SD = 0.7) vs. 2.2 (SD = 0.7), *p* < 0.05]. The cohorts were similar to each other on remaining health, putative barriers to ART, and whether care was received in a hospital clinic or not. (Data not shown on a table for parsimony.)

### Factors associated with readiness to initiate ART

As noted above (see Individual/attitudinal-level factors), readiness for ART was low, although there was great variability. In bivariate analyses, readiness for ART was not associated with cohort (clinic vs. peer) or any socio-demographic or health/health history variable. Instead, a number of individual/attitudinal and social-level factors were predictive. Physician mistrust (*B* = −10.4) was negatively associated with readiness for ART at a statistically significant level, while self-efficacy beliefs (*B* = 5.5), positive outcome expectancies (*B* = 6.3), favorable beliefs about the personal necessity of ART (*B* = 17.5), and positive internal norms (*B* = 7.9) were positively associated with readiness for ART. In Table 4, we present a correlation matrix of the variables found to be associated with readiness for ART. Correlations with ART readiness show medium to large effect sizes (73) and relationships are in the expected direction. In a multiple regression of ART readiness on the five variables with a significant bivariate association, only ART self-efficacy (*B* = 3.6) and personal necessity beliefs (*B* = 11.54) had significant unique associations with ART readiness.

**Table 4:** **Correlations among variables associated with ART readiness**.

	1	2	3	4	5	6
ART readiness (0–100)	1.00					
Physician mistrust (0–4)	−0.22*	1.00				
ART self-efficacy beliefs (0–10)	0.34**	−0.24*	1.00			
ART outcome expectancies (0–10)	0.48**	−0.33**	0.36**	1.00		
Personal necessity for ART (0–4)	0.46**	−0.20	0.15	0.69**	1.00	
Internal norms in support of ART (0–6)	0.30**	−0.26*	0.28**	0.51**	0.50**	1.00

*p < 0.05; **p < 0.01.

## Discussion

The present study provides a detailed exploration of a vulnerable and hard-to-reach population, PLHA-NOA, and extends past research on PLHA-NOA to include those not embedded in hospital clinic settings. Indeed, diverse recruitment and engagement approaches are necessary for this population, given that at least half of PLHA are estimated to not engage in HIV care at the recommended frequency (3). We focused on African-American and Latino/Hispanic individuals in the present study because they comprise the majority of PLHA and experience a unique set of barriers to ART, partly rooted in culture. Indeed, understanding these barriers can inform efficient, effective, and culturally targeted interventions (15).

### Feasibility of reaching PLHA-NOA and lessons learned

Contrary to expectations, we found that it was challenging to recruit PLHA-NOA in clinic settings in the context of this study, but more feasible to do so using a peer-recruitment method. Several factors impeded recruitment in the hospital HIV clinics. First, most PLHA in these settings (>95%) were taking ART, highlighting the success of these high-quality care settings in engaging patients and bringing them on ART in a timely fashion. Yet among those in clinics not on ART, patterns of infrequent attendance at medical care appointments greatly reduced their chances of participation, in part because providers were unable to confirm the patient’s appropriateness for the study and because they did not present for recruitment. Further, when they did present for recruitment, these PLHA were often disinterested in discussing ART use, in many cases, expressing fear of and anxiety related to doing so. This suggests that novel strategies to engage wary patients are needed. Peer-driven interventions may be one promising approach. In peer-driven intervention, the initial contact is made by a peer rather than a study or clinic staff member (74, 75). This can foster engagement because peers tend to have a high level of credibility (74, 75). Study findings also suggest that self-selection and/or structural barriers reduce the numbers of PLHA with the most serious barriers to ART in clinic settings; these individuals may feel more comfortable in non-medical community-based settings, and non-HIV settings such as harm reduction organizations. The present study also provides important information on whether PLHA-NOA are socially networked with each other. The rapid rate of peer-recruitment suggests that large numbers of them are socially networked, which provides the basis for future studies of this population using social network recruitment approaches. Our study findings further suggest that clinic settings can draw on outreach approaches to engage PLHA-NOA in HIV care at appropriate levels and address barriers to ART.

The present study was consistent with existing literature on hospital clinic vs. community-based settings in some respects (49, 50): PLHA-NOA recruited from the clinics tended to have better health status indicators, even when not taking ART. Yet perhaps in contrast to this past literature, those in non-clinic settings were presented to the study in later stages of HIV disease (although we do not know when they first engaged with the health care setting). Overall, the present study indicates that PLHA recruited through peers, most of whom are not embedded in HIV hospital clinics, are farther along in the course of their HIV disease and have greater contextual risk factors than those in clinics.

We found that an in-depth screening process fostered engagement with the study and, overall, more accurate reporting of ART status. This study component can be retained in the future research. Yet we also found it challenging to confirm medical information from the providers of a substantial number of the peer-recruited cohort, mainly because they were poorly engaged in care. Yet confirmation of HIV health status (CD4, viral load) and ART status (taking vs. not taking) is vital to studies such as these. Indeed, PLHA-NOA commonly report that communication with providers is challenging (76). It is possible that a small number of participants in the present study were taking ART but did not report it to the study in order to enroll. Future studies can consider evaluating CD4 and viral load directly (that is, not relying on the medical record), and recent ART use can be assessed through hair samples (77), to more definitely assess HIV indicators and recent ART use patterns.

### Reasons for ART discontinuation

Among those who had taken ART in the past, participants tended to discontinue their regimens for reasons that appear largely preventable or addressable. Participants did not report difficulties obtaining ART or ART prescription refills, highlighting the strength of the local HIV care system in proving patients with access to ART. Instead, contextual changes (e.g., life circumstances changed, lost housing), substance use, and factors suggesting a low level of readiness for ART (e.g., changed my mind, was not ready) contributed to ART discontinuation, particularly among those in the peer-recruited cohorts. This suggests that interventions and programs in clinical settings to increase readiness for ART may also promote sustained ART use (78). Side effects were the single most common reason for discontinuation, consistent with past research (79, 80). Yet participants can be prepared for side effects through interventions to improve coping skills for self-management (81) or training on mindfulness based stress reduction (82). Participants who initiated ART some time ago may not realize that the tolerability of regimens has improved, and patient education on the side effect profiles of newer regimens may boost ART uptake (83). Yet PLHA from racial/ethnic minority backgrounds, particularly older PLHA, vividly recall the early days of the HIV epidemic when AZT (Zidovudine) was given in high doses, resulting in side effects including anemia, neutropenia, hepatotoxicity, and myopathy (84). Thus for PLHA from racial/ethnic minority backgrounds, an understanding of ART side effects may be interpreted through a filter of suspicion and medical distrust that is more common among people from racial/ethnic minority backgrounds than Whites, as we have described above (see Introduction) (20). This suggests that interventions to increase ART uptake acknowledge this cultural history and, importantly, address the emotions associated with ART use, particularly fear, in addition to providing health education and fostering adherence skills. Indeed, the role of fear of ART in the problem of PLHA-NOA cannot be over-emphasized (51, 85). In future research, we will use quantitative and qualitative research to better understand the issue of side effects, including how PLHA learn about side effects, which side effects cause the greatest concerns, and what types of intervention approaches can help PLHA manage their fear of side effects and the side effects themselves.

### Multi-level barriers to ART

An understanding of barriers to ART can inform the design of programs to improve ART uptake with high adherence. The literature suggests that PLHA-NOA have low levels of HIV knowledge and largely negative attitudes toward ART. Although our ability to interpret study data is somewhat limited by the lack of published norms, findings suggest that knowledge of HIV treatment was at least adequate (average 69% items correct), albeit with room for improvement, and attitudes toward ART ranged from somewhat negative to neutral. As might be expected in a sample of PLHA-NOA, readiness to initiate ART was low [mean 36.2 (SD = 34.4) on a 0–100 point scale], but with substantial variability. Overall, participants in the two cohorts evidenced similar rates of barriers to ART, although those in the peer-recruited cohort reported greater perceived need for ART, perhaps consistent with their inferior health status and lower rates of concern about/fear of ART, compared to those recruited from clinics.

### Factors associated with readiness to initiate ART

Readiness to initiate ART has been found in a number studies to predict treatment outcomes and adherence, and may be an important target for interventions to improve ART uptake. We did not find that socio-demographic or health factors were associated with readiness in this sample. A number of attitudes and beliefs were associated with greater readiness, including higher levels of trust in one’s physician, self-efficacy, outcome expectancies, perceived personal need for ART, and positive perceived internal norms (how one feels about others taking ART). This suggests that attitudes toward ART are complex and multifaceted, and include individual and social aspects, and that these various aspects of ART beliefs must be considered together to improve readiness for ART and ART uptake. Horne and colleagues (86) found that uptake of ART was associated with perceptions of personal necessity and concerns about potential adverse effects, and these factors predicted subsequent adherence, independent of clinical variables and depression (86). The present study suggests a number of other social-cognitive factors that may influence these behaviors. Counseling approaches such as motivational interviewing are useful to “unpack” complex and ambivalent attitudes, and thereby enhance intrinsic motivation for health behavior change (54).

### Those who have never taken ART previously (treatment naïve)

We found that PLHA who had never taken ART in the past, called “treatment naïve PLHA,” differ from those with past ART experience. These treatment naïve PLHA tended to present with a pattern of more serious barriers to ART than their ART-experienced peers, including being less knowledgeable about ART, and evidencing greater fears of ART and of ART-related stigma. Future intervention efforts can take into consideration that younger PLHA and those who have lived with HIV for shorter periods of time may require more support in order to initiate and benefit from ART, in comparison to their peers who have had more time to adapt to their HIV diagnoses and gain knowledge about ART. Yet we do not know at present whether treatment naïve PLHA are less likely than their experienced peers to initiate ART, a question which can be explored in the future research.

### ART initiation cannot be disentangled from adherence

The problem of delayed ART initiation cannot be considered separately from that of ART adherence. Despite recent advances in ART regimens’ tolerability and efficacy, and potent new technologies to monitor adherence, the problem of fostering long-term adherence to ART remains one of the greatest challenges facing the public health field today (87, 88). A large body of literature has examined the effects of social/behavioral interventions to improve ART adherence with respect to medication-taking behavior and clinical outcomes (87–92). A number of promising adherence interventions had emerged in the last decade, evidencing effects on adherence behavior and viral load (93–96). Yet a review by Simoni and colleagues found that while overall adherence interventions increased the chances of high ART adherence compared to controls, the effect on clinical outcomes (viral load in this case) was not statistically significant, and these effects tend to be short-lived (91). Further, many adherence interventions found successful in experimental trials may not be feasible for implementation in community-based settings and real-world clinics (88). In a recent meta-analysis, de Bruin and colleagues found that nonwhite patients had a lower chance of achieving undetectable viral load compared to whites. However, they did not find racial/ethnic differences in adherence success rates. They speculated these viral load differences may have been caused by pre-treatment clinical status, or that adherence differences were not found due to a lack of adherence measures with cross-cultural validity (90). Thus efforts to develop behavioral interventions to improve ART initiation can benefit from and build on this rich literature in the domain of ART adherence, with particular attention to non-white PLHA, who may experience the greatest barriers to adherence and/or good clinical outcomes.

### Limitations

The present study’s limitations include a lack of knowledge of the true size and scope of this vulnerable population, as well as the relatively small sample sizes which may have prevented us from detecting differences between cohorts. Further, the convenience sampling approach may limit the generalizability of study findings to other settings. In addition, the low proportion of clinic-recruited PLHA suggests that those enrolled may not be representative of the larger population of PLHA in clinics; they may represent those most willing to consider ART, thus biasing findings toward more favorable outcomes. Moreover, the ability of the present study to examine complex relationships across multiple variables was limited by sample size. Structural equation modeling in larger samples is therefore needed to better understand processes that promote or impede readiness for ART. Last, the present study was exploratory in nature and targeted PLHA able to conduct research activities in English. We will extend future research to mono-lingual Spanish-speaking PLHA, although the size of this population appears small in our local area (<5% of PLHA) (97).

### Implications for future research and practice

To achieve the goals of the National HIV/AIDS Strategy (NHAS), PLHA must initiate ART in a timely fashion, and remain on ART with high adherence through their lives. Studies on the HIV continuum of care indicate that improvements are needed at each stage, and the CDC calls for particular efforts to reduce health disparities by race and ethnicity (3). The present study advances our knowledge of a vulnerable and hard-to-find population of PLHA that is critical to efforts to reduce gaps along the HIV continuum of care. Importantly, the results of this study and recent conceptualizations of the HIV continuum of care in low-resource, international settings (98) strongly suggest that engagement in care and use of ART are not static, but entail ongoing, multi-faceted processes. Future work is needed to articulate a more detailed HIV continuum of care with a focus on intermediate steps between linkage to care, engagement in care, ART initiation, and ART continuation with high adherence, and devise intervention strategies for improving outcomes along this more detailed care continuum.

## Conflict of Interest Statement

The authors declare that the research was conducted in the absence of any commercial or financial relationships that could be construed as a potential conflict of interest.
